# HERNIIA-II trial (Hernia Endoscopic oR opeN repair In chIldren Analysis): a protocol of a multicentre randomised controlled trial to study the (cost-)effectiveness of laparoscopic hernia repair compared to open hernia repair in children 0–16 years

**DOI:** 10.1136/bmjopen-2025-110662

**Published:** 2025-12-04

**Authors:** Sanne C Maat, Lore Elise de Vreeze, Roxanne Eurlings, Johannes Anema, Robertine Van Baren, Jasper V Been, Frank van den Broek, Hamit Cakir, Johanna M van Dongen, Floris Ferenschild, Jurgen C de Graaff, Robert Nijveldt, Anne Ottenhof, Arianne J Ploeg, Hester Rippen, Jetske Ruiterkamp, Jos W R Twisk, Erik Vermeulen, Ralph De Wit, Gerda Zijp, Ernest L W van Heurn, Joep P M Derikx

**Affiliations:** 1 Paediatric Surgery, Emma Kinderziekenhuis Amsterdam UMC, Amsterdam, The Netherlands; 2 Universiteit van Amsterdam, Amsterdam Gastroenterology Endocrinology Metabolism, Amsterdam UMC Locatie AMC, Amsterdam, The Netherlands; 3 Amsterdam Reproduction and Development research institute, Amsterdam UMC Locatie AMC, Amsterdam, The Netherlands; 4 Department of Paediatric Surgery, MosaKids Children’s Hospital, Maastricht University Medical Centre, Maastricht, The Netherlands; 5 Public and Occupational Health, EMGO+ Institute for Health and Care Research Public and Occupational Health, Amsterdam, The Netherlands; 6 Department of Paediatric Surgery, University Medical Centre Groningen Beatrix Childrens Hospital, Groningen, The Netherlands; 7 Department of Paediatrics, Division of Neonatology, Sophia Children’s Hospital, University Medical Centre Rotterdam, Rotterdam, The Netherlands; 8 Department of Surgery, Maxima Medical Centre, Veldhoven, The Netherlands; 9 Department of Pediatric Surgery, MosaKids Children’s Hospital, Maastricht University Medical Centre, Maastricht, The Netherlands; 10 Department of Health Sciences, Faculty of Science, Amsterdam Public Health Research Institute, Vrije Universiteit Amsterdam, Amsterdam, The Netherlands; 11 Department of Paediatric Surgery, Amalia Children’s hospital, Radboud University Medical Centre, Nijmegen, The Netherlands; 12 Department of Anesthesiology, Weill Cornell Medicine, New York, New York, USA; 13 Department of Surgery, Isala Hospital, Zwolle, The Netherlands; 14 Department of Surgery, Flevoziekenhuis, Almere, The Netherlands; 15 Surgery, Alrijne Zorggroep, Leiderdorp, The Netherlands; 16 Kind en Ziekenhuis, Utrecht, The Netherlands; 17 Department of Pediatric Surgery, Wilhelmina Children’s Hospital, University Medical Centre Utrecht, Utrecht, The Netherlands; 18 Department of Epidemiology and Data Science, Amsterdam UMC, Amsterdam, The Netherlands; 19 Department of Surgery, Spaarne Gasthuis, Haarlem, The Netherlands; 20 Department of Surgery, Medisch Spectrum Twente, Enschede, The Netherlands; 21 Paediatric Surgery, Haga Hospital Juliana Childrens Hospital, Den Haag, The Netherlands; 22 Department of Pediatric Surgery, Amsterdam Reproduction and Development Research Institute, Amsterdam UMC Location AMC, Amsterdam, The Netherlands; 23 Amsterdam Gastroenterology Endocrinology Metabolism, Amsterdam UMC Locatie VUmc, Amsterdam, The Netherlands; 24 Department of Paediatric Surgery, Emma Childrens’ Hospital UMC, Amsterdam, The Netherlands

**Keywords:** PAEDIATRIC SURGERY, PAEDIATRICS, Randomized Controlled Trial

## Abstract

**Introduction:**

Inguinal hernia repair is one of the most frequently performed operations in the paediatric population and can be performed according to two approaches: open or laparoscopic. At present, decisive evidence about the best treatment strategy is lacking and consequently, there is an ongoing debate about the most (cost-)effective treatment for the paediatric inguinal hernia. The aim of the Hernia Endoscopic oR opeN repair In chIldren Analysis—trial (HERNIIA2-trial) is to estimate the (cost-)effectiveness of the laparoscopic percutaneous internal ring suturing (PIRS) technique compared with open repair in children aged 0–16 years with a primary unilateral inguinal hernia.

**Methods and analysis:**

A national multicentre randomised controlled trial will be performed including 464 children aged 0–16 years with a primary unilateral inguinal hernia. Patients will be randomised between the open or PIRS technique. The primary outcome is the number of reoperations within 2 years after primary surgery. Secondary outcome measures are: operative and postoperative complications, total duration of surgery, postoperative pain, length of admission, time to normal daily activities, cosmetic appearance of the scar, social and healthcare costs and health-related quality of life. Furthermore, cost-effectiveness will be assessed from a societal and healthcare perspective.

**Ethics and dissemination:**

The protocol was approved by the ethics committee of the Amsterdam University Medical Hospital. Informed consent will be obtained by parents and, if possible, according to age, by patient. The study will be conducted according to the principles of the Declaration of Helsinki (2013) and in accordance with the Medical Research Involving Human Subjects Act (WMO) and Good Clinical Practice. Study findings will be disseminated through scientific publications, conferences and patient-friendly materials. The national study network of participating centres will facilitate rapid dissemination and implementation within the Netherlands and potentially abroad.

**Trial registration number:**

ClinicalTrials.gov PRS (ID NCT06451432).

**STRENGTHS AND LIMITATIONS OF THIS STUDY**

- The Hernia Endoscopic oR opeN repair In chIldren Analysis trial is a multicentre randomised controlled trial that compares laparoscopic percutaneous internal ring suturing inguinal hernia repair with open inguinal hernia repair in the paediatric population.
- This study will provide decisive evidence regarding the most (cost-)effective treatment strategy for the paediatric inguinal hernia: open or laparoscopic hernia repair.

## Introduction

 The incidence of paediatric inguinal hernia ranges from 0.8% to 5% and increases to more than 30% in preterm born infants.^1 2^ Surgical treatment is necessary because of the risk of incarceration of bowel, testis or ovary, which occurs in approximately 3–16% of unoperated children with inguinal hernia.^2 3^ Approximately 6–8% of all children develop a metachronous contralateral inguinal hernia (MCIH) after unilateral inguinal hernia repair. This requires second surgery and thus a second hospital admission and exposure to anaesthesia, which in turn results in additional psychological stress for both children and parents, increased healthcare costs and potential complications of repeated anaesthesia in children. Open inguinal hernia repair is the most commonly performed treatment strategy in children; however, the laparoscopic approach is increasingly used in developed countries.^4^ Laparoscopic paediatric hernia repair allows visualisation of both inguinal regions, without making an extra incision, thereby enabling detection of a contralateral patent processus vaginalis (CPPV). If a CPPV is present, it can be closed simultaneously during laparoscopy to prevent the possible development of an MCIH. However, laparoscopic hernia repair is performed under general anaesthesia whereas open hernia repair offers the possibility for loco-regional (spinal) anaesthesia, which might be beneficial as repeated general anaesthesia carries risks for near critical incidents.^5 6^ The technique for open inguinal hernia repair is similar worldwide. In contrast, over the past two decades, multiple laparoscopic techniques have been developed, broadly categorised as intracorporeal or extracorporeal techniques. The difference between these techniques is the number of trocars (typically three ports) and the placement of the suture knot. Intracorporeal techniques use an intra-abdominal knot, whereas the extracorporeal technique uses a subcutaneous knot to close the inguinal hernia.

Although inguinal hernia repair is the most commonly performed operation by paediatric surgeons, there remains a lack of consensus regarding the most superior technique in children who need to undergo inguinal hernia repair: open or laparoscopic hernia repair.^7^ In adults, laparoscopic inguinal hernia repair is advised, because it results in less postoperative pain and faster recovery compared with open repair.^8^ However, results for adults cannot be adopted for children since paediatric inguinal hernia has a different aetiology. Paediatric inguinal hernia is a congenital disorder and thus always a lateral hernia. A paediatric inguinal hernia develops from a processus vaginalis that did not close during development instead of muscle weakness of the lower abdominal wall in adults. Therefore, an inguinal hernia in children can be repaired primarily instead of using mesh repair.

We recently performed a meta-analysis including all currently available randomised controlled trials (RCTs) comparing open and laparoscopic paediatric inguinal hernia repair.^9^ This meta-analysis consisted of eight RCTs (n=733), of which five compared open with laparoscopic intracorporeal suturing and three compared open with laparoscopic extracorporeal suturing. The results of our meta-analysis showed that there were no differences when comparing the open and laparoscopic intracorporeal suturing technique regarding complications (OR 0.50, 95% CI 0.14 to 1.79), recurrence rate (OR 0.88, 95% CI 0.20 to 3.88), MCIH rate (OR 0.28, 95% CI 0.04 to 1.86) and unilateral operation time (weighted mean difference 0.62, 95% CI −0.579 to 6.95). Similar findings are reported by three other recent meta-analyses even though they also included studies with other study designs than RCTs.^10^,^14^ However, the laparoscopic extracorporeal technique resulted in fewer complications and a shorter operative time compared with the open technique.^9^ The superiority of the extracorporeal hernia repair is also supported by our most recent systematic review (15 studies, n=3680 children) that compared intracorporeal and extracorporeal laparoscopic closing techniques in children.^15^ The review showed that single-port extracorporeal closure results in less recurrent hernias (OR 0.11, 95% CI 0.02 to 0.48) and a shorter operation time compared with the intracorporeal technique. Another benefit of the extracorporeal technique is that, in contrast to intracorporeal laparoscopic techniques, it does not require intra-abdominal suturing, which is time consuming, has a high learning curve and carries a higher risk for iatrogenic visceral injury following multiple manipulations with instruments in the peritoneal cavity.^16 17^

Percutaneous internal ring suturing (PIRS) technique is the least invasive laparoscopic technique that uses extracorporeal suturing and has a similar complication rate to other laparoscopic techniques.^15^ To complete the learning curve for this technique, a surgeon needs to perform 30–35 surgeries, which is supposed to be shorter than the learning curve for the intracorporeal suturing technique.^18 19^ This technique requires only one umbilical port and a puncture point access through which the suture is placed. In conclusion, the PIRS technique leads to visualisation of the whole peritoneal cavity without leaving any extra scars and is a safe laparoscopic hernia repair technique, even if it is performed by a surgeon with a basic skill level in endoscopic surgery.^16 18^ Therefore, the PIRS technique seems to be the preferable laparoscopic technique for the repair of a paediatric inguinal hernia.

The 2019 national guideline of the Dutch Society of Surgeons and the 2021 international European Paediatric Surgical Association guideline regarding the treatment of paediatric inguinal hernia stated that there was no sufficient high-level evidence to draw a conclusion about the best treatment strategy.^7 20^ To date, no large RCT has compared open repair with the laparoscopic PIRS technique. Consequently, there is an ongoing debate about the best treatment strategy for inguinal hernia repair in children, and decisive evidence on the superiority of either one of those treatment strategies is lacking. Execution of large RCTs, which take into account all relevant outcome measures, the use of one laparoscopic technique and costs, is required to obtain homogenous results to decide on the superiority of one of either treatment strategy. The aim of this RCT is to extensively assess the effectiveness and cost-effectiveness of laparoscopic PIRS technique compared with open hernia repair in children aged 0 months–16 years with a primary unilateral inguinal hernia.

### Protocol report

This trial protocol was reported in accordance with the Standard Protocol Items: Recommendations for Interventional Trials 2013 Statement.^21^

## Methods: participants, interventions and outcomes

### Study setting

This trial will be a national multicentre trial initiated by the paediatric surgery department of the Emma Children’s Hospital of Amsterdam University Medical Centre. The trial will be conducted across 12 participating centres including both university and general hospitals: Emma Children’s Hospital, Amsterdam University Medical Centre; Alrijne Hospital; Amalia Children’s Hospital, Radboud University Medical Centre; Beatrix Children’s Hospital, University Medical Centre Groningen; Flevo Hospital; Isala Hospital; Juliana Children’s Hospital, Haga Hospital; Máxima Medical Centre; Medical Spectrum Twente; Mosa Kids Children’s Hospital, Maastricht University Medical Centre; Spaarne Gasthuis; Wilhelmina Children’s Hospital; University Medical Centre Utrecht. Patient characteristics and data on reported outcome measures will be registered in an electronic case record form using Castor Electronic Data Capture (EDC).^22^

### Eligibility criteria

Children aged 0 months–16 years with a primary unilateral inguinal hernia, undergoing hernia repair are eligible to participate in this study. A potential subject who meets any of the following criteria will be excluded from participation in this study: children with (1) incarcerated inguinal hernia, which has to be operated on immediately, (2) recurrent or metachronous inguinal hernia, (3) ventricular-peritoneal drain, (4) non-descended testis, (5) parents who are not able to understand the nature or consequences of the study.

### Study design

**Figure 1:**
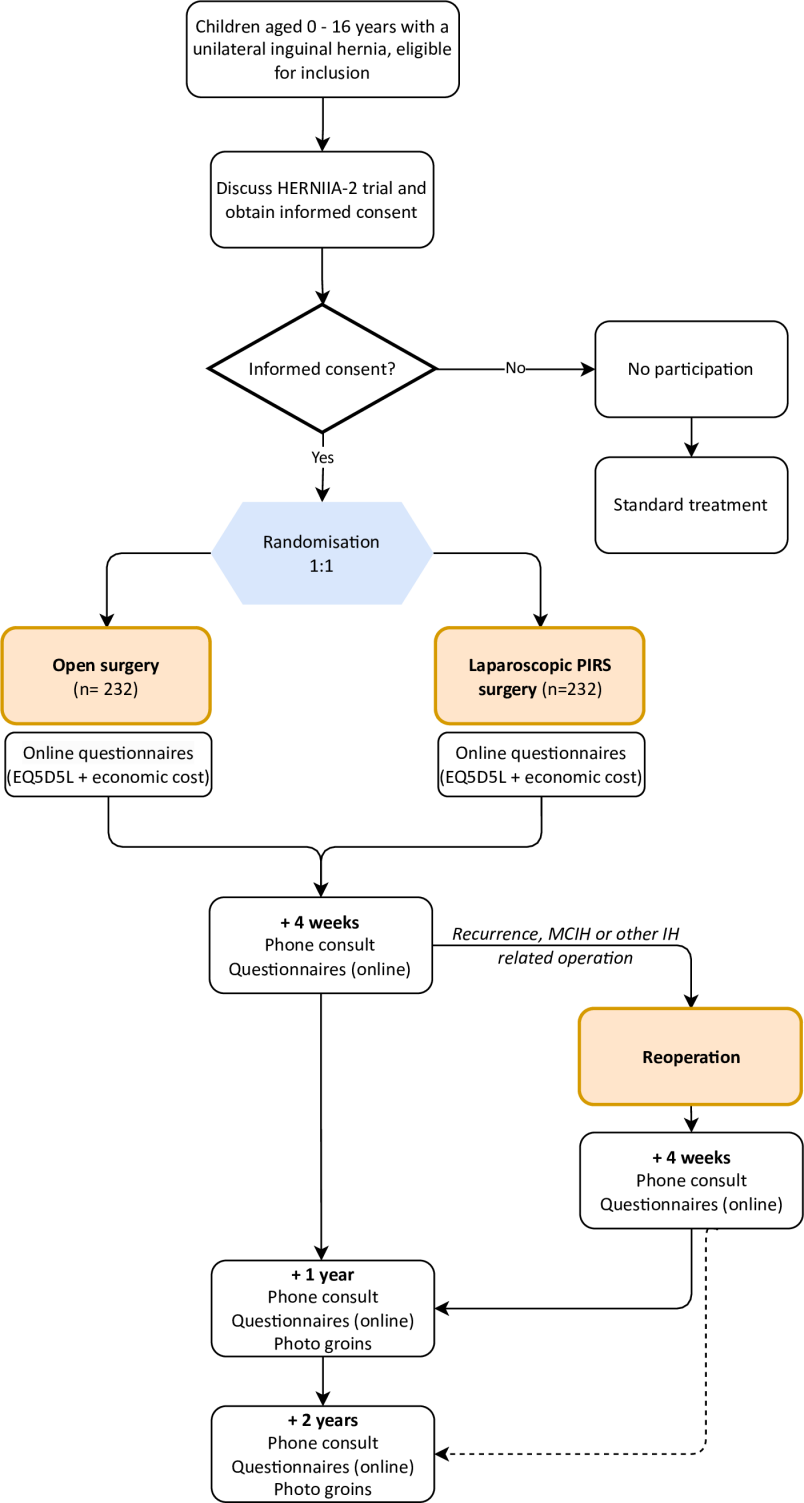
Flow chart of participants included in the study. HERNIIA-2, Hernia Endoscopic oR opeN repair In chIldren Analysis; IH, inguinal hernia; MCIH, metachronous contralateral IH, PIRS, percutaneous internal ring suturinercutaneous Internal Ring Suturing. EuroQoL 5-Dimension 5-Level (EQ5D5L)

An RCT will be performed and 464 children with a primary unilateral inguinal hernia will be randomised (1:1) to either open repair or laparoscopic PIRS repair (figure 1). All operations will be performed by experienced (laparoscopic) paediatric surgeons.

All children with a unilateral inguinal hernia visit the outpatient clinic of the (resident/fellow) paediatric surgeon with their parents/caretakers. The surgeon explains the study, hands over the information letter and informed consent form to the parents/caretakers and asks permission to hand over their contact details to the research team. All (resident/fellow) surgeons that will be involved in the conduct of this clinical trial will receive training in order to provide information to the parents/caretakers, according to the standard operating procedure (SOP) and Good Clinical Practice (GCP) guidelines. Most children are operated on within 3–4 weeks after their outpatient clinic visit. After a minimum of 7 days, a medical researcher will call the parents/caretakers to ask if they will participate in this study and if so, ask them to sign the two informed consent letters. If, however, surgery is scheduled within 1 week of the outpatient clinic visit, the researcher will contact the parents after a minimum of 24 hours to allow sufficient time for questions and consideration of participation. Parents will be asked to return the signed informed consent forms by post. After receiving these forms, a member of the research team will sign them, provide parents with an original copy and randomise the patient using a web-based application. If returning the forms by post is not feasible within the required time, a member of the research team will collect the informed consent forms on the day of admission and sign both forms. One informed consent form will be distributed to the parents/caretakers. An example of the informed consent form is available in the onlinesupplemental files 1  2.

4 weeks, 1 year and 2 years after the primary inguinal hernia surgery, all children (or their parents/caretakers) will be called by a member of the research team. Prior to the 1-year and 2-year call, patients or their parents/caretakers will be asked to send a photo of the subject’s groin. The latter is done to score testicular atrophy, recurrence or contralateral hernia. See figure 1 for the complete participant timeline. It is not possible to blind children nor their parents/caretakers or healthcare professionals for the study arm, since different scars will develop and instructions for the postoperative treatment of the wound(s) have to be given.

### Relevant concomitant care

Concomitant surgery such as repair of an umbilical hernia or draining a hydrocele component is permitted during the trial and will be registered.

### Primary outcome

Primary outcome measure of this RCT will be: number of children requiring a reoperation, related to hernia repair, within 2 years after primary surgery.

### Secondary outcomes

Secondary outcome measures are: operative (ie, injury of spermatic vessels or spermatic cord, tuba lesions, bleeding and apnoea) and postoperative complications (ie, haematoma/scrotal oedema, hydrocele, wound infection, iatrogenic ascent of the testis, testicular atrophy and ileus), total duration of surgery (including anaesthesia and overall time spent in operating room), postoperative pain, length of hospital stay, time to normal daily activities, cosmetic appearance, social and healthcare costs and health-related quality of life (HRQOL). All outcome measures will be measured within 2 years after the primary inguinal hernia repair.

Furthermore, cost-effectiveness will be assessed from a societal and healthcare perspective and will include cost(s) of surgery, hospitalisation, control visits, reoperation, complications and loss of productivity of the parents/caretakers. Most resource use data will be acquired from medical records, for which specific data entry forms will be used. Data on productivity losses of the parents/caretakers will be collected by online questionnaires before surgery and at 4 weeks, 1 year and 2 years after primary surgery. In cases of recurrence or MCIH, additional questionnaires will be completed before and 4 weeks after reoperation. The cost of the operation will be estimated using a bottom-up micro costing approach. Other costs will be valued using guideline prices.

### Participant timeline

#### Sample size

Reoperation includes both MCIH and recurrence rates. Since recent studies showed that the recurrence rate is similar between the open and laparoscopic group (0.3 vs 0.2%),^23^ we used reoperation rates due to MCIH to calculate our sample size. The latest International Paediatric Endosurgery Group guidelines state that the MCIH rate after open repair is 6–8%. We expect that the laparoscopic repair reduces the MCIH rate to 1% since the CPPV will be closed during the same procedure. Thus, reoperation based on MCIH is performed in approximately 6% of the patients in the open group and in approximately 1% in the laparoscopic PIRS group. We consider a reduction of 5% points of children requiring reoperation clinically relevant.

A total sample size of 422 patients is needed to detect such a reduction with a power of 0.80 at a two-sided alpha of 0.05 (nQuery Advisor V.7.0). Taking into account 10% loss to follow-up, we need to include 464 children.

#### Assignment of intervention

Randomisation will be performed using Castor EDC, a web-based application that employs a computer-generated list with random block sizes of four or six, which will be rendered by an independent data manager. The block size of the randomisation scheme is unknown to the study personnel. Participants and investigators are aware of allocation; masking is impossible because of the nature of the intervention.

## Methods: data collection, management and analysis

### Data collection methods and management

Data will be collected in an electronic case record form using Castor EDC. We will collect the following baseline characteristics from the participants: gestational age, birth weight, age at day of surgery, weight at day of surgery, duration of surgery, duration of hospital stay, sex, side of the hernia, comorbidity, CPPV rate, complications (wound infection, testicular atrophy, apnoea, recurrence) and development of contralateral hernia.

To ensure the privacy of the patients, all the participants’ data will be encoded using a five-digit number that is only used for this study (XX-XXX). This encoding will be as follows: the first two digits are for the participating centre; the second three digits are for the consecutive patient number in that centre. Only the principal investigator, the coordinating investigator and the project leader will have access to the encoding key which links the code to the participants’ personal data. After termination of the study, participants will have full access to their results and data will be explained to them when requested from the researcher at their site. This researcher can obtain the results via the coordinating investigator or the project leader. In case this study (by coincidence) reveals any findings important to participants, the principal investigator makes sure the attending doctor will be informed.

In order to protect participant privacy, all data will be encoded and accessible only to the principal investigator, coordinating investigator and project leader. The data collected from all centres will be stored at Amsterdam UMC for a period of 15 years.

Before the execution of any future additional analyses not mentioned in the current protocol, an amendment will be handed in to the Medical Ethical Review Committee (METC) for ethical approval.

### Statistical methods

Data will be assessed using IBM SPSS Statistics V.28. All statistical testing will be performed two-sided with α=0.05 and according to the intention-to-treat principle.

#### Primary study parameter

Primary outcome is the proportion of reoperations within 2 years after primary hernia repair. The number and percentage of reoperations will be reported for both treatment groups.

A Fisher Exact test will be performed and the absolute risk difference and OR with their 95% CIs will be calculated. In addition, a logistic regression analysis will be performed adjusted for centre and possible confounders (sex, gestational age, age at time of surgery and initial hernia side). The main effect estimate will be the adjusted OR with the corresponding 95% CI.

#### Secondary study parameters

Secondary outcomes include operative and postoperative complications, total duration of surgery and anaesthesia, postoperative pain, length of hospital stay, time to normal daily activities, cosmetic appearance, social and healthcare costs and HRQOL.

Mean (SD) or median (IQR) differences and corresponding 95% CIs will be calculated for length of hospital stay, duration of surgery and anaesthesia, time to normal daily activities, postoperative pain and cosmetic appearance. OR together with the 95% CI will be calculated for operative and postoperative complications. Linear and logistic regression analysis will be performed to adjust for centre and possible confounders (sex, gestational age at birth and initial hernia side). The CPPV rate will only be described for the laparoscopic group, since the open repair does not allow for contralateral exploration.

Differences in HRQOL will be reported using medians and IQRs at the following time points: preoperatively (baseline) and at 4 weeks, 1 year and 2 years after the primary hernia repair. If applicable, differences in HRQOL scores will also be reported prior to and 4 weeks after reoperation.

Missing values will be handled using multiple imputation. The imputed datasets will be analysed separately, and the results will then be pooled according to Rubin’s rules.

#### Economic evaluation

Cost-effectiveness analyses are performed according to the intention-to-treat principle. For the economic evaluation, missing data will again be handled using multiple imputation. To deal with the highly skewed nature of cost data, 95% CIs around the differences in costs will be estimated using the bias corrected and accelerated bootstrap method, with 5000 replications. Incremental cost-effectiveness ratios (ICERs) will be calculated by dividing the difference in costs by those in quality adjusted life years (QALYs) and ‘disease-specific’ physical functioning. QALYs will be estimated by administering the EuroQol 5-Dimension 5-Level (EQ-5D-5L) questionnaire at preoperative and postoperative time points (4 weeks, 1 year and 2 years) and valuing the participants’ health states using the Dutch tariff.^24^ To graphically illustrate the uncertainty surrounding the ICERs, bootstrapped incremental cost-effect pairs will be plotted on cost-effectiveness planes. A summary measure of the joint uncertainty of costs and effects will be presented using cost-effectiveness acceptability curves, which indicate the probability of an intervention being cost-effective in comparison with the control condition for a range of willingness-to-pay values (ie, the maximum amount of money decision-makers are willing to pay to gain one extra unit of effect). Various sensitivity analyses will be performed to assess the robustness of the results.

## Methods: monitoring

### Data monitoring

Monitoring will be performed by an independent monitor provided by Amsterdam UMC. The monitor will verify reported data, ensure that the study is performed according to the METC-approved protocol and monitor the well-being of patients included in the study. For further details, the monitoring plan can be consulted.

An interim analysis assessing the primary endpoint will be performed by the research team after 1 year of follow-up to assess the effectivity of the laparoscopic PIRS technique versus open repair. In the case of a significant difference between two groups, an analysis will be performed to see if the study can continue with a subgroup. If no significant difference is found during the interim analysis, the study will continue as stated in this protocol.

### Harms

If the health or safety of subjects is in jeopardy, the sponsor (initiating centre Amsterdam UMC) will suspend the study based on section 10, subsection 4 of the Medical Research Involving Human Subjects Act (WMO). When such action is taken, the sponsor will, without delay, inform the METC and provide the reason why the study is (temporarily) stopped. Without a positive decision of the METC, the study will not continue.

The definition of adverse events in this study is the experience of any undesirable effect by a subject during the length of the study, whether or not considered related to the study procedure. All adverse effects reported by the subject/parents/caretakers or observed by the investigator will be recorded.

We do not expect to have any suspected unexpected serious adverse reactions; therefore, we deem registration of such events as not applicable.

All adverse events will be followed up until they have diminished, disappeared or at least a stable situation has been reached. Depending on the event, additional medical procedures (treatment, referral) may be needed.

### Patient and public involvement

This project collaborates with the Child & Hospital Foundation (Stichting Kind & Ziekenhuis, K&Z), a Dutch organisation dedicated to improving paediatric care and promoting patient participation in hospital policy, as well as with the client boards of the participating hospitals. K&Z and the hospital client boards act as patient representatives, advising and informing policymakers, healthcare professionals and researchers. Patients and parents have been actively involved in the study from the outset, contributing to the design and the selection of relevant patient-centred outcome measures. Throughout the trial, they will continue to be engaged in monitoring progress, advising on unexpected developments and codeveloping dissemination and implementation materials aimed at patients and families following data analysis.

## Ethics and dissemination

### Ethics approval

The study will be conducted according to the principles of the Declaration of Helsinki (2013) and in accordance with the WMO and GCP. Ethical approval has been obtained from the institutional review board of VU University Medical Centre in Amsterdam, The Netherlands (registration number: 2020.340). Additional local ethics approvals have been granted by the medical ethical review boards of all participating centres. Before enrolment of participants in this study, written informed consent will be obtained from the parents/caretakers.

### Adverse events reporting

All adverse events that have occurred within the first four postoperative weeks will be reported and followed until they have abated, or until a stable situation has been reached. In accordance with section 10, subsection 4, of the WMO, the sponsor will suspend the study if there is sufficient ground that continuation of the study will jeopardise subject health or safety. The sponsor will notify the accredited METC without undue delay of a temporary halt, including the reason for such an action. The study will be suspended pending a further positive decision by the accredited METC. The investigator will take care that all subjects are kept informed.

### Safety committee

Both treatment strategies, laparoscopic (intervention) and open (control) hernia repair, are currently performed in children with inguinal hernia who need to undergo repair. Consequently, there are no additional risks for subjects of this study and it is therefore not necessary to install a data safety monitoring board. Because this study is undertaken in children, the investigators want to install a safety committee, which can be asked for help if a serious adverse event is encountered. The committee will be formed by a paediatric surgeon and a paediatrician.

### Dissemination plan

All results, positively or negatively, will be presented at (inter)national conferences and published in peer reviewed (inter)national journals. In the case of rejection of results by peer reviewed journals, the results will be made public in a trial registry or a database. A clinical trial agreement will be signed by involved parties. The national study network of participating centres will facilitate rapid dissemination and implementation within the Netherlands and potentially abroad.

### Trial status

Official initiation of the study commenced on 1 February 2024, after final ethical approval was granted. The first participant was enrolled on 3 June 2024, and enrolment will continue until the required sample size has been reached. The current protocol is V.1.8 date 10 January 2025 (online supplemental file 3). We expect to complete the inclusion of patients end of 2026. The study will be considered complete at the time of the last patient’s last visit, which marks the 2-year follow-up and is expected at the end of 2028.

## Supplementary material

10.1136/bmjopen-2025-110662 online supplemental file 1

10.1136/bmjopen-2025-110662 online supplemental file 2

10.1136/bmjopen-2025-110662 online supplemental file 3
