# Supplementary material for: HERNIIA-II trial (Hernia Endoscopic oR opeN repair In chIldren Analysis): a protocol of a multicentre randomised controlled trial to study the (cost-)effectiveness of laparoscopic hernia repair compared to open hernia repair in children 0–16 years
Source: BMJ Open. 2025 Dec 4;15(12):e110662. doi: 10.1136/bmjopen-2025-110662 (PMC12684125; doi:10.1136/bmjopen-2025-110662)
Supplement: online supplemental file 1 [file bmjopen-15-12-s001.pdf]

Information form 12-16 years

HERNIIA 2 trial

Dear patient,

Are you interested in participating in a groin hernia study? Here, you can read more information about the study and your rights. Read this carefully so you will know what to decide about. You may take your time to think before making a decision.

Your parents will also receive information about this study. You can discuss the study with them, and together, you and your parents will make a decision.

Questions and contact details

Do you have questions? Discuss them with your parents. Or ask the researcher. You can write down your questions below.

You can also call/email the researcher anytime:

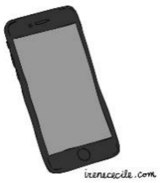

Lore de Vreeze

l.e.devreeze@amsterdamumc.nl of 06-50100868

Would you like to talk to a doctor about the study that is not involved in this study? Call or email then with: Dr. Tim de Meij, ******************

Space to write down your questions:

Tip: Take a picture of your questions, you will have them with you when talking to the doctor/researcher.

About the study

This research is conducted at AUMC. A total of 464 children are participating. The study has been reviewed and approved. The name of the committee responsible for the evaluation is called: METC VUMC.

Why this study?

There are two methods to repair a groin hernia in children: open surgery or minimal invasive surgery. The purpose of this study is to determine whether groin hernia repair is best performed through laparoscopic surgery or the open method.

Background:

When a child has a hernia, it needs to be operated. This is necessary to to prevent damage to the intestines, ovaries (in girls), or spermatic cord (in boys). Hernia repair can be done through the open method, involving a small incision in the groin, or through laparoscopic surgery.

In the Netherlands, hernia repair is traditionally done through the open method. In many other countries, it is done using laparoscopic surgery, where a camera is inserted through a small incision below the navel. The open method has been used for a long time and is a technique with very few complications. Laparoscopic surgery is a newer technique in which the entire abdomen can be viewed using a camera. Both methods have been shown to be safe and effective in previous studies, but it's not clear which treatment is better. The goal of this study is to determine whether a hernia is best repaired through laparoscopic surgery or the open method. The study will also examine other outcomes, such as the duration of the operation, length of hospital stay, and the appearance of scars.

How to participate?

There are 2 groups in the study: one group undergoes the open method, and the other group undergoes laparoscopic surgery. We draw lots to decide in which group you will be assigned. This assignment cannot be chosen, and the doctor has no influence over it. We will tell you in which group you have been assigned.

During the doctor's visit, we will decide whether it is possible to participate in the study. This will take approximately 10 minutes. This includes a physical examination and questions about your health.

You will visit the hospital a total of two times, regardless of participation in the study: one visit before the surgery and another visit for the surgery itself.

Your parents will receive a questionnaire about your health and a cost questionnaire: before the operation, 4 weeks, and 1 year after the operation. You and your parents can fill these in together. Also, you and your parents will be called 4 weeks, 1 year, and 2 years after the surgery to discuss how you are doing.

About the treatment

As mentioned earlier, you will be operated by using the open method or through laparoscopic surgery. We do not know which technique is better. The potential **advantage** of laparoscopic surgery is the ability to examine the other groin without making an additional incision. This occurs in 1 out of 10 children, and it is not visible from the outside. If a groin hernia is seen on the other side, it is also repaired during the same operation. The **disadvantage** is that complications may occur in both operated groin hernias, such as wound infection, bleeding, damage to the blood supply to the testicle (in boys),

and recurrence of the groin hernia after surgery. The surgical risks of both laparoscopic surgery and the open method are very small.

Important to know:

- You cannot participate in another study at the same time.

Compensation

- You do not get compensation for participating.

You rights

Do you have to participate?

No, you can **decide for yourself** whether you want to participate. If you do not want to participate, you will not have to, even if your parent's prefer that you do. If you want to participate, you can sign the form. Even after that, **you can still stop anytime** you want. Just let the researcher know if you decide to stop. You don't have to explain why you want to stop.

If you choose not to participate, you will get the standard treatment we normally use for repairing a groin hernia, which is the open method through a small incision in the groin.

Withdrawal of consent

If you want to stop, tell the researcher. This is called: withdrawing your consent. The information that has already been collected will still be used for the research.

Your data

For the study, we need two things that belong to you:

- ***Personal data*** = information about who you are, such as your date of birth and where you live.

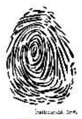

- ***Medical data:*** (also a type of personal data) information about your health, such as whether you are sick and whether you use medication.

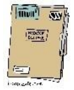

The data (personal and medical) will be given a code, such as letters and numbers. This way, someone else cannot immediately see that the data is yours. Only the researcher knows which code corresponds to whom. Other people only see the code, so they do not know your name.

These **two things are needed for conducting the research**. Your parents give permission for us to use this information. If you want to know more about what exactly we do with your data, ask your parents, as it is explained in their information letter. You can also ask the researcher.

Insurance

There is insurance for this research, and it is mandatory. If you want to know more about it, you can ask your parents. It is further explained in the information letter for parents. You can also ask the researcher.

Your decision

The form

Do you want to participate? If so, you can sign the consent form. We also need a signature from you parents/guardian.

Want to know more?

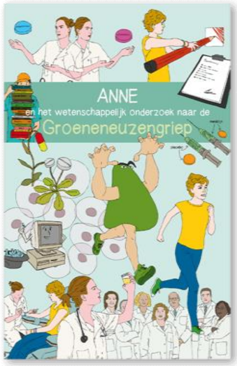

Do you want to know more about research and you rights?

Visit www.kindenonderzoek.nl

On this website you will also find the comic 'Anne en de Groeneneuzengriep' about research.

Attachment:

1. **Consent form for minor participants aged 12 t/m 16 years**

Attachment 1: Consent form for minor participants aged 12 t/m 16 years

- I have **understood** the information. I could also ask questions. My **questions have been answered**.
- I had **enough time to decide** whether I want to participate.
- I know that I am **not obligated** to participate.
- I understand that I **can always stop** if I no longer wish to participate.

I want to participate in this study.

Name participant:

Signature: Date : __ / __ / __

__**This part is fort he researcher:**__

I declare that I have fully informed this participant about the mentioned research.

If information becomes known during the study that could influence the participant's consent, I will inform him/her promptly.

Name researcher (or representative):

Signature: Date: __ / __ / __

The participant receives a complete information letter, along with a signed version of the consent form.

Attachment 1: Consent form for minor participants aged 12 t/m 16 years

- I have **understood** the information. I could also ask questions. My **questions have been answered**.
- I had **enough time to decide** whether I want to participate.
- I know that I am **not obligated** to participate.
- I understand that I **can always stop** if I no longer wish to participate.

I want to participate in this study.

Name participant:

Signature: Date : __ / __ / __

<u>**This part is fort he researcher:**</u>

I declare that I have fully informed this participant about the mentioned research.

If information becomes known during the study that could influence the participant's consent, I will inform him/her promptly.

Name researcher (or representative):

Signature: Date: __ / __ / __

The participant receives a complete information letter, along with a signed version of the consent form.
