# Supplementary material for: HERNIIA-II trial (Hernia Endoscopic oR opeN repair In chIldren Analysis): a protocol of a multicentre randomised controlled trial to study the (cost-)effectiveness of laparoscopic hernia repair compared to open hernia repair in children 0–16 years
Source: BMJ Open. 2025 Dec 4;15(12):e110662. doi: 10.1136/bmjopen-2025-110662 (PMC12684125; doi:10.1136/bmjopen-2025-110662)
Supplement: online supplemental file 2 [file bmjopen-15-12-s002.pdf]

Information for Parents/Caregivers of study participants for medical scientific research

Does your child have a groin hernia?
Groin hernia in children: A study comparing two repair methods (HERNIIA II trial).

Dear Sir/Madam,

You are receiving this letter because your child* is undergoing groin hernia surgery. We would like to invite you and your child to participate in a research study. Participation is voluntary. To participate, your written consent is required. Before deciding whether to participate, we hereby provide an explanation of the study. Please read this information carefully and feel free to ask the researcher for clarification if you have any questions. You can also discuss it with your partner, friends, or family. General information about participating in a study can be found on the website: www.rijksoverheid.nl/mensenonderzoek.
* of degene wie u vertegenwoordigt.

General Information
This study is conducted by the VUmc in Amsterdam, and the Medical Ethical Review Committee of VUmc has approved this research.

Objective of the Study
When a child has a groin hernia, surgery is necessary to prevent damage to the intestines or - in girls, the ovaries or - in boys, the spermatic cord. Groin hernia repair can be done through an open method or a laparoscopic (minimally invasive) procedure. We aim to investigate whether a laparoscopic approach is more effective in groin hernia repair than the open method.

Background of the Study
In the Netherlands, groin hernia repair is traditionally performed using the open method through an incision in the groin. However, nowadays, hernia repair is increasingly performed with a laparoscopic procedure. In this approach, the surgeon inserts a camera through a small incision below the umbilicus. The surgeon closes the hernia sac with a stitch inserted through the skin into the abdominal wall.

The open method has been used for a long period of time with few complications. The laparoscopic procedure is a newer technique allowing the surgeon to view the entire abdomen internally. Both techniques are currently performed, and the choice between them depends on the surgeon's and parents' preferences. We anticipate that the laparoscopic procedure may lead to fewer complications, such as damage to the spermatic cord in boys or blood supply to the testicle, and in girls, damage to the ovary. This is because the surgeon has a clear view of the abdominal structures during the laparoscopic procedure. Additionally, during the same laparoscopic surgery, we can examine the other side of the groin and repair a possible hernia on that side as well, which occurs in about 10% of children and is not visible externally. Previous studies indicate that both the open method and laparoscopic procedure are safe and effective treatments, but it is unclear which

treatment is superior. The goal of this research is to determine whether hernia repair is best achieved through a laparoscopic procedure or the open method.

What does it mean if your child participates in the study?

Assessment

First, we determine if your child can participate. The doctor/researcher will confirm the presence of a groin hernia through physical examination or photo imaging. The researcher will also inquire about any other diseases or conditions your child may have.

Treatment/Surgery

All children will undergo surgery for a groin hernia. In one group (half of the participating children), the groin hernia will be repaired through a laparoscopic procedure via a small incision below the belly button. In the second group, the groin hernia will be repaired using the open technique, involving a small incision in the groin. Randomization determines the group your child will be in, and the doctor has no influence over this.

Visits and Measurements

You and your child will visit the hospital twice. These visits are no different from those if you were not participating in this study. During the visits, the following will occur:

- During one visit, we will conduct a physical examination.
- During one visit, we will perform the surgery.

Furthermore, the researcher will call you four weeks, one year, and two years after the surgery to inquire about your child's well-being and whether any complications have occurred. Additionally, we ask you to complete questionnaires before the surgery, 4 weeks, and 1 year after the surgery. One questionnaire focuses on your child's health, and the other examines the costs you incurred, such as work absenteeism due to caring for your child. These questionnaires can be filled out on paper or online. You can complete them at home, and each time it will take about 10 minutes in total.

What is different from usual care?

This study does not involve extra hospital visits. Unlike usual care, we will call you after four weeks and two years, and we request you to complete a set of questionnaires both before and after the surgery.

What is expected from you

If your child participates in this study, he/she cannot participate in another medical scientific study simultaneously. It is essential that you contact the researcher:

- If your child is admitted to or treated in a hospital, even if it is for something else.
- If your child develops sudden health issues.
- If you no longer want your child to participate in the study.
- If your contact information changes.

What are the possible complications
The recovery of a groin hernia through either the laparoscopic or open method carries the same risks. Fortunately, these risks do not occur frequently, but it is uncertain whether one of the two techniques has fewer risks. This is also something we will investigate.

These complications can occur, although infrequently:
- Infection of the wound;
- Recurrence of the operated groin hernia;
- Damage to the blood supply to a testicle (in boys).

If during the laparoscopic procedure a groin hernia is also found on the other side, it will be repaired. In both groins, the above complications can occur.

What are the possible pros and cons of participating in this study
Participation in this study does not directly benefit or harm your child. Your child contributes to gaining more knowledge on how to best treat groin hernias in children. Therefor we can improve the care for children with groin hernias. The cons of participating in this study include the extra time and effort it takes. We will call you three times over 2 years, and we ask you to complete questionnaires. Filling out the questionnaire will take about 3 times 10 minutes.

The main advantage of *the laparoscopic procedure* is that if a groin hernia is found on the other side, it can be repaired immediately without making an extra incision.

Sometimes, other things can accidentally be discovered during the study. If this happens, it will be discussed with you. If you prefer not to know about such incidental findings, your child can not participate in the study.

What happens if your child resists study participation?
If your child resists (does not cooperate) during the study, the researcher must stop immediately. It is sometimes challenging to precisely define what resistance is. This will be discussed with you before the study begins. The researcher will adhere to the Code of Conduct for resistance in minors.

If you do not want to participate or want to stop the study participation
You will decide whether your child participates in the study. Participation is voluntary and you will be given sufficient time (at least five days) to consider whether you and your child want to participate. If you choose not to participate, your child will get the usual treatment for a groin hernia.

If you initially agree to participate but change your mind later, you can withdraw at any point during the study. Your child will then get the usual treatment for a groin hernia.. You do not have to provide a reason for withdrawing, but you must inform the researcher. The data collected until that point will be used for the research.

If there is new information about the study that is crucial for you, the researcher will inform you. You will have the option to decide whether you want to continue participating.

End of the study
Your participation in the study will end if:
- You choose to stop;
- The researcher decides it is better for you and your child to stop
- The medical ethics review committee, the government or the hospital decides to stop the study.

The entire study is finished when all participants are done. After processing all the data, the researcher will inform you about the most important outcomes of the study.

Use and storage of your and your child's data
For this study, medical and personal data from your child will be collected and used. Collecting, using, and storing this data is necessary to answer the questions posed in this study. To protect the privacy, the data will be assigned a code, omitting your child's name and identifiable information. Only with the code key, the data be traced back to you. The code key remains securely stored in the local research institution. In reports and publications about the study, the data will not be traceable to your child.

Your and your child's data
All your child's data will remain confidential. Only the principal investigator, study coordinator, and the project leader know the code assigned to your child.

Some individuals may have access to your child's medical and personal data to ensure the study is conducted properly and reliably. Those with access include: the research team, the safety committee overseeing the study, an auditor hired by the study's executor and the Health Care Inspectorate. They will keep your child's data confidential. By signing the consent form, you authorize the collection, storage, and access to your child's medical and personal data. Researchers at VUmc will keep your data for 15 years.

More information about your rights regarding data processing
For general information about your rights regarding the processing of your personal data, you can visit the website of the Dutch Data Protection Authority (Autoriteit Persoonsgegevens). If you have questions about your rights, you can contact the person responsible for processing your personal data. For this study, it is Amsterdam UMC, location AMC. See Annex A for contact details.

For questions or complaints about the processing of your personal data, we recommend contacting the research location first. You can also contact the Data Protection Officer of the institution (for contact details, refer to Annex A) or the Dutch Data Protection Authority.

Research registration
This study is also listed in a registry of medical scientific research, namely https://clinicaltrials.gov/. This website does not contain information that can be traced back to your child. However, the website may display a summary of the results. You can find this study under the name: HERNIIA-2 trial.

Is there an insurance coverage when your child participates in the study?
Insurance has been obtained for everyone participating in this study. The insurance covers potential harm caused by the research. Not all types of harm are covered. More information about the insurance can be found in **Annex B**, including details on where to report any damages.

Information for general practitioner and/or treating specialist
We always send a letter to your general practitioner and/or treating specialist to inform them that your child is participating in the study. Sometimes, we may also request medical records from your general practitioner and/or treating specialist for your child's safety. If you are not comfortable with this, your child cannot participate in the study.

Are there any costs or compensations involved when you child participates in the study?
Participation in this study does not involve any additional costs for you. You and your child will not receive any reimbursement or payment for participating in this research.

Do you have any questions?
If you have any questions, you can contact your child's treating physician or the principal investigator at the AMC. For independent advice on participating in this study, you can consult an independent physician. They are knowledgeable about the research but have no affiliation with it. For complaints, it is advisable to contact the complaints committee of your hospital. All contact details can be found in **Annex A:** Contact Information.

Thank you very much for your attention.

Attachments to this information
A. Contact details Amsterdam UMC, location AMC
B. Information about the insurance
C. Consent form for parents/guardians

Annex A: contact details for Amsterdam UMC, VU medisch centrum

Principal investigator: Dr. Joep Derikx, Department of Pediatric Surgery, Amsterdam UMC, location AMC, Meibergdreef 9. 1105 AZ, Amsterdam, E-mail: j.derikx@amsterdamumc.nl, Phone: 020 566 9111.
In case of emergencies, the on-duty Surgical Assistant in our hospital can be contacted 24 hours a day on phone number 020 566 9111.

Independent doctor: Dr. Tim de Meij, Department of Paediatric Gastroenterology, Amsterdam UMC, VUmc, De Boelelaan 1117, 1081 HV Amsterdam, E-mail: ************, Phone: 020 444 5350.

Complaints: Klacht Complaints can be addressed to the Patient & Care Provider Service Center. E-mail: klachtenfunctionaris@amc.nl, or Phone: 020 566 3350.
Data Protection Officer:
Data Protection Officer AMC: fg@amc.uva.nl

Annex B: information about the insurance

For everyone participating in this research, VU University Medical Center has secured insurance. The insurance covers damages resulting from participation in the research. This applies to damages occurring during the research or within four years after its completion. Damages must be reported to the insurer within those four years. The insurance does not cover all damages. Below is a brief overview of damages that are not covered. These provisions are outlined in the Decision on Compulsory Insurance for Medical Scientific Research involving Human Subjects (Besluit verplichte verzekering bij medisch-wetenschappelijk onderzoek met mensen). This decision can be found on www.ccmo.nl, the website of the Central Committee on Research Involving Human Subjects (See 'Bibliotheek' and then 'Wet- en regelgeving').

In case of damage, you can contact your treating physician or directly get in touch with the insurer.

The insurer fort his study is (Polisnummer: 624.529.204):

Name:	Onderlinge Waarborgmaatschappij Centramed B.A.
Address:	Postbus 7374, 2701 AJ Zoetermeer
Phone number:	070 301 70 70
E-mail:	info@centramed.nl

The insurance provides coverage of €650.000 per participant and €5.000.000,- for the entire study (and €7.500.000,- per year for all studies from the same sponsor).

The insurance does **not** cover the following damages:

- Damage resulting from a risk about which you have been informed in the written information. This exception does not apply if the risk occurs more severely than anticipated or if the risk was highly improbable;
- Damage to your health that would have occurred even if you had not participated in the study;
- Damage resulting from not (fully) following instructions or guidelines;
- Damage to your descendants, as a consequence of any adverse effects of the study on you or your descendants;
- Damage resulting from an existing treatment method in research on existing treatment methods;

Annex C: Consent Form: Does your child have a groin hernia?

I have been asked to give consent for the participation of the following person/my child in this medical-scientific research:

Naam of participant (Child): Date of birth: __ / __ / __

- I have read the information letter. I could also ask questions. My questions have been adequately answered. I had enough time to decide if I want my child to participate.
- I know that participation is voluntary, and I can decide at any time that my child will not participate. I do not need to provide a reason for this.
- I give consent to inform the general practitioner/specialist(s) treating my child that my child is participating in this research. I also give consent to obtain information from the general practitioner/specialist(s) treating my child.
- I am aware that some people involved in the oversight of the research may access my child's data. These individuals are listed in this information letter.
- I give consent for the collection and use of data to answer the research question in this study.
- I give consent for my data and my child's data to be sent to a central location (this central location, or research site, is located at VUmc).
- I give consent to retain my data and my child's data at the research site for 15 years after this study.
- I agree that my child participates in this research. We ask you to have **both parents** sign this form. If single custody, please check below.

Name parent/guardian:
Signature: Date: __ / __ / __
Name parent/guardian:
Signature: Date: __ / __ / __

☐ Single custody has been indicated by the undersigned.

I hereby declare that I have fully informed the above person(s) about the mentioned research. If during the research, information becomes known that could influence the consent of the parent or guardian, I will promptly notify them.

Researcher’s name (or representative):
Signature: Date: __ / __ / __

Additional information provided by (if applicable):
Name: Function:
Signature: Date: __ / __ / __

The participant receives a complete information letter, along with a copy of the signed consent form.

Annex C: Consent Form: Does your child have a groin hernia?

I have been asked to give consent for the participation of the following person/my child in this medical-scientific research:

Naam of participant (child): Date of birth: __ / __ / __

- I have read the information letter. I could also ask questions. My questions have been adequately answered. I had enough time to decide if I want my child to participate.
- I know that participation is voluntary, and I can decide at any time that my child will not participate. I do not need to provide a reason for this.
- I give consent to inform the general practitioner/specialist(s) treating my child that my child is participating in this research. I also give consent to obtain information from the general practitioner/specialist(s) treating my child.
- I am aware that some people involved in the oversight of the research may access my child's data. These individuals are listed in this information letter.
- I give consent for the collection and use of data to answer the research question in this study.
- I give consent for my data and my child's data to be sent to a central location (this central location, or research site, is located at VUmc).
- I give consent to retain my data and my child's data at the research site for 15 years after this study.
- I agree that my child participates in this research. We ask you to have **both parents** sign this form. If single custody, please check below.

Name parent/guardian:
Signature: Date: __ / __ / __
Name parent/guardian:
Signature: Date: __ / __ / __
☐ Single custody has been indicated by the undersigned.

I hereby declare that I have fully informed the above person(s) about the mentioned research. If during the research, information becomes known that could influence the consent of the parent or guardian, I will promptly notify them.

Researcher’s name (or representative):
Signature: Date: __ / __ / __

Additional information provided by (if applicable):
Name: Function:
Signature: Date: __ / __ / __

The participant receives a complete information letter, along with a copy of the signed consent form.
