# Supplementary material for: HERNIIA-II trial (Hernia Endoscopic oR opeN repair In chIldren Analysis): a protocol of a multicentre randomised controlled trial to study the (cost-)effectiveness of laparoscopic hernia repair compared to open hernia repair in children 0–16 years
Source: BMJ Open. 2025 Dec 4;15(12):e110662. doi: 10.1136/bmjopen-2025-110662 (PMC12684125; doi:10.1136/bmjopen-2025-110662)
Supplement: online supplemental file 3 [file bmjopen-15-12-s003.pdf]

Open vs Laparoscopic hernia repair in children: A randomized controlled trial

(January 2025)

Subtitle:
*HERNIIA II trial: '**H**ernia **E**ndoscopic o**R** ope**N** repair **I**n ch**I**ldren **A**nalysis.*

Writing committee

[redacted]

PROTOCOL TITLE 'Open vs Laparoscopic hernia repair in children: A randomized controlled trial'

**Protocol ID**	HERNIIA 2020
**Short title**	HERNIIA II trial
**EudraCT number**	Not applicable
**Version**	1.8
**Date**	25-11-2024
**Coordinating investigator**<br><br>**Project leader**	[redacted]
**Principal investigator(s) (in Dutch: hoofdonderzoeker/ uitvoerder)**	[redacted]
Sites: (investigator names and contact details; Appendix 1)	Amsterdam UMC, location VU University Medical Centre, Amsterdam, the Netherlands.

	Amsterdam UMC, location Amsterdam Medical Centre, Amsterdam, the Netherlands.<br>Flevoziekenhuis, Almere, the Netherlands<br>Isala Ziekenhuis, Zwolle, the Netherlands<br>UMC Groningen, Groningen, the Netherlands<br>Maastricht UMC+, Maastricht, the Netherlands<br>Haga ziekenhuis, the Hague, the Netherlands<br>Alrijne Ziekenhuis, Leiderdorp, the Netherlands<br>Maxima Medisch Centrum, Veldhoven, the Netherlands<br>Erasmus MC, Rotterdam, the Netherlands<br>Medisch Spectrum Twente, Twente, the Netherlands<br>Radboud UMC, Nijmegen, the Netherlands
**Sponsor (in Dutch: verrichter/opdrachtgever)**	Amsterdam UMC, location VU Medical Centre, Amsterdam, the Netherlands.
**Subsidising party**	Not applicable
**Independent expert (s)**	[redacted]
**Laboratory sites**	Amsterdam UMC, location Amsterdam Medical Centre, Amsterdam, the Netherlands.

PROTOCOL SIGNATURE SHEET

Name	Signature	Date
**Head of department:** [redacted]		
**Principal Investigator** dr. J.P.M. Derikx (paediatric) surgeon, Emma Children's Hospital, Amsterdam UMC, University of Amsterdam & Vrije Universiteit, Amsterdam, the Netherlands.<br><br>**Project leader:**<br><br>drs. L.E. de Vreeze, PhD candidate Emma Children's Hospital, Amsterdam UMC, University of Amsterdam & Vrije Universiteit, Amsterdam, the Netherlands.<br><br>.		

TABLE OF CONTENTS

1. INTRODUCTION AND RATIONALE 10
2. OBJECTIVES 12
3. STUDY DESIGN 13
4. STUDY POPULATION 14
4.1 Population (base) 14
4.2 Inclusion criteria 14
4.3 Exclusion criteria 14
4.4 Sample size calculation 14
5. TREATMENT OF SUBJECTS 15
5.1 Investigational product/treatment 15
5.2 Use of co-intervention (if applicable) 16
5.3 Escape medication (if applicable) 16
6. INVESTIGATIONAL PRODUCT 17
7. NON-INVESTIGATIONAL PRODUCT 17
8. METHODS 18
8.1 Study parameters/endpoints 18
8.1.1 Main study parameter/endpoint 18
8.1.2 Secondary study parameters/endpoints (if applicable) 18
8.1.3 Other study parameters (if applicable) 19
8.2 Randomisation, blinding and treatment allocation 19
8.3 Study procedures 20
8.4 Withdrawal of individual subjects 20
8.4.1 Specific criteria for withdrawal (if applicable) 21
8.5 Replacement of individual subjects after withdrawal 21
8.6 Follow-up of subjects withdrawn from treatment 21
8.7 Premature termination of the study 21
9. SAFETY REPORTING 21
9.1 Temporary halt for reasons of subject safety 21
9.2 AEs, SAEs and SUSARs 21
9.2.1 Adverse events (AEs) 21
9.2.2 Serious adverse events (SAEs) 22
9.2.3 Suspected unexpected serious adverse reactions (SUSARs) 22
9.3 Annual safety report 22
9.4 Follow-up of adverse events 23
9.5 Safety Committee 23
10. STATISTICAL ANALYSIS 24
10.1 Primary study parameter(s) 24
10.2 Secondary study parameter(s) 24
10.3 Other study parameters 24
10.4 Interim analysis (if applicable) 25
11. ETHICAL CONSIDERATIONS 25

11.1 Regulation statement 25
11.2 Recruitment and consent 25
11.3 Objection by minors or incapacitated subjects (if applicable) 26
11.4 Benefits and risks assessment, group relatedness 26
11.5 Compensation for injury 27
11.6 Incentives (if applicable) 27
12. ADMINISTRATIVE ASPECTS, MONITORING AND PUBLICATION 28
12.1 Handling and storage of data and documents 28
12.2 Monitoring and Quality Assurance 28
12.3 Amendments 29
12.4 Annual progress report 29
12.5 Temporary halt and (prematurely) end of study report 29
12.6 Public disclosure and publication policy 29
13. STRUCTURED RISK ANALYSIS 29
14. APPENDIX 1
REFERENCES 31

LIST OF ABBREVIATIONS AND RELEVANT DEFINITIONS

**ABR**	**General Assessment and Registration form (ABR form), the application form that is required for submission to the accredited Ethics Committee; in Dutch: Algemeen Beoordelings- en Registratieformulier (ABR-formulier)**
**AE**	**Adverse Event**
**AR**	**Adverse Reaction**
**CA**	**Competent Authority**
**CCMO**	**Central Committee on Research Involving Human Subjects; in Dutch: Centrale Commissie Mensgebonden Onderzoek**
**CV**	**Curriculum Vitae**
**CPPV**	**Contralateral patent processus vaginalis**
**DSMB**	**Data Safety Monitoring Board**
**EU**	**European Union**
**EudraCT**	**European drug regulatory affairs Clinical Trials**
**GCP**	**Good Clinical Practice**
**GDPR**	**General Data Protection Regulation; in Dutch: Algemene Verordening Gegevensbescherming (AVG)**
**IB**	**Investigator's Brochure**
**IC**	**Informed Consent**
**IMP**	**Investigational Medicinal Product**
**IMPD**	**Investigational Medicinal Product Dossier**
**MCIH**	**Metachronous contralateral inguinal hernia**
**METC**	**Medical research ethics committee (MREC); in Dutch: medisch-ethische toetsingscommissie (METC)**
**PIRS**	**Percutaneous Internal Ring Suturing**
**(S)AE**	**(Serious) Adverse Event**
**SPC**	**Summary of Product Characteristics; in Dutch: officiële productinformatie IB1-tekst**
**Sponsor**	**The sponsor is the party that commissions the organisation or performance of the research, for example a pharmaceutical company, academic hospital, scientific organisation or investigator. A party that provides funding for a study but does not commission it is not regarded as the sponsor, but referred to as a subsidising party.**
**SUSAR**	**Suspected Unexpected Serious Adverse Reaction**
**UAVG**	**Dutch Act on Implementation of the General Data Protection Regulation; in Dutch: Uitvoeringswet AVG**

**WMO**	**Medical Research Involving Human Subjects Act; in Dutch: Wet Medisch-wetenschappelijk Onderzoek met Mensen**

SUMMARY

Rationale: Paediatric inguinal hernia repair is one of the most frequently performed operations in children, with an incidence ranging from 0.8 to 5% that increases to more than 30% in preterm born infants[1, 2]. Treatment is necessary because of the risk of incarceration of bowel, testis or ovary, which occurs in approximately 3-16% of children with inguinal hernia[2, 3]. Open inguinal hernia repair is the most performed treatment strategy in children, however, laparoscopic inguinal hernia repair in children is increasingly performed as it allows easy contralateral inspection and potentially results in shorter operation time and fewer complications. Nowadays, there are many laparoscopic techniques of which, in our opinion, the Percutaneous Internal Ring Suturing (PIRS) technique is the most preferable. The PIRS technique is the least invasive laparoscopic technique that uses extra-corporeal suturing and has a similar complication rate to other laparoscopic techniques. High-level evidence regarding the superiority of PIRS technique versus open hernia repair is lacking in children.

Objective: To study the effectiveness and a cost-effectiveness analysis of laparoscopic PIRS technique compared to open hernia repair in infants aged 0 months to 16 years of age with a primary unilateral inguinal hernia

Study design: A randomized controlled trial. Infants that need to undergo inguinal hernia repair will be randomized to either open or laparoscopic correction.

Study population: Infants aged between 0 months and 16 years who will undergo hernia repair of a primary unilateral hernia.

Intervention (if applicable): Open hernia repair or laparoscopic Percutaneous Internal Ring Sutering (PIRS) repair

Main study parameters/endpoints: Primary endpoint: Number of operations related to inguinal hernia within one year after inguinal hernia repair. Other endpoints: Duration of surgery, operative and postoperative complications, duration of hospital admission, postoperative pain, time to full recovery, CPPV-rate, cosmetic appearance and cost-effectiveness (health care and social costs). **Nature and extent of the burden and risks associated with participation, benefit and group relatedness:** Both the open and PIRS technique are commonly used techniques in paediatric inguinal hernia repair and are dependent of the surgeon performing the procedure. Therefore, no extra burden of risk exist regarding this study.

Both treatment strategies are currently performed in children with unilateral sainguinal hernia who need to undergo hernia repair. Consequently, there are no additional risks for subjects of this study and it is therefore not necessary to install a DSMB.

1. INTRODUCTION AND RATIONALE

The incidence of paediatric inguinal hernia ranges from 0.8 to 5% and increases to more than 30% in preterm born infants (1, 2). Treatment is necessary because of the risk of incarceration of bowel, testis or ovary, which occurs in approximately 3-16% of children with inguinal hernia (2, 3). Open inguinal hernia repair is the most performed treatment strategy in children; however, the laparoscopic approach is increasingly used in current practice (4). Although inguinal hernia repair is the most commonly performed operation by paediatric surgeons, there still is no clear consensus which technique is superior in children who need to undergo inguinal hernia repair: open or laparoscopic hernia repair.

Laparoscopic hernia repair allows better visualization of the inguinal region thereby enabling detection of a contralateral patent processus vaginalis (CPPV), which can be simultaneously closed since the presence of a CPPV might result in development of a metachronous contralateral inguinal hernia (MCIH). Open hernia repair offers the possibility for loco regional (caudal) anaesthesia, which might be beneficial as repeated or prolonged (more than three hours) general anaesthesia carries risks for near critical incidents and the U.S. Food and Drug Administration (FDA) recently released a warning that repeated or prolonged (more than three hours) general anaesthesia potentially harms the child's developmental brain(5, 6).

Over the past twenty years multiple laparoscopic techniques were developed, broadly categorized in intra- or extracorporeal techniques. These techniques varied in number of trocars, type of suturing and type of suturing materials. We recently performed a meta-analysis including all currently available randomized controlled trials comparing open and laparoscopic hernia repair (7). This meta-analysis consisted of eight trials (n=733) of which 5 compared open with laparoscopic intra-corporeal suturing and 3 that compared open with laparoscopic extra-corporeal suturing. The results of our meta-analysis showed that there were no differences when comparing the open and laparoscopic intra-corporeal suturing technique. However, subgroup analysis showed that the extra-corporeal technique resulted in less complications and a shorter operative time compared to the open technique (7).

Percutaneous Internal Ring Suturing (PIRS) technique is the least invasive laparoscopic technique that uses extra-corporeal suturing and has a similar complication rate to other laparoscopic techniques. Therefore, PIRS is the most preferable technique. The PIRS technique requires only one umbilical port and a puncture point access through were the suture is placed. In contrary to intra-corporeal laparoscopic techniques, the PIRS technique does not require intra-abdominal suturing, which is time consuming, has a high learning curve and carries a higher risk for iatrogenic visceral injury following the many manipulations with instruments in the peritoneal cavity (8, 9). In conclusion, the PIRS technique leads to visualization of the peritoneal cavity without leaving any scars and is a safe laparoscopic

hernia repair technique, even if it is performed by a surgeon with a basic skill level in endoscopic surgery (8, 10).

Several randomized controlled trials comparing laparoscopic with open paediatric hernia repair have been published, but many outcome parameters have not been addressed which include among others some peri- and post-operative complications (such as apnea, ileus) and health care costs (11). Furthermore, there is no current randomized controlled trial comparing open and laparoscopic PIRS technique. Consequently, there is an ongoing debate about the best treatment strategy and decisive evidence on the superiority of one of either treatment strategies is lacking. The aim of this randomized controlled trial is to provide an extensive comparison for effectiveness and a cost-effectiveness analysis of laparoscopic PIRS technique compared to open hernia repair in infants aged 0 months to 16 years of age with a primary inguinal hernia.

2. OBJECTIVES

This randomized controlled trial studies the difference between open and laparoscopic PIRS repair with total of operations related to inguinal hernia within one year after inguinal hernia repair as primary outcome measure. Other endpoints are duration of surgery, operative and postoperative complications, use of different anaesthetic techniques, duration of hospital admission, postoperative pain, time to full recovery, CPPV-rate, cosmetic appearance and cost-effectiveness (health care and social costs).

3. STUDY DESIGN

A randomized controlled trial will be performed and 464 infants with a primary unilateral inguinal hernia will be randomized to either open repair or laparoscopic PIRS repair. All operations will be performed by experienced (laparoscopic) paediatric surgeons.

One and two years after surgery all children will be called by the researcher. Prior to this call, patients or their parents will be asked to send a photo of the subject groins.. The latter is done to score testicular atrophy, recurrence or contralateral hernia. This will also be scored during a video call two years post-surgery. It is not possible to blind children or their parents/caretakers or health care professionals for the study arm, since different scars will develop and instructions have to be given for the postoperative treatment of the wound(s).

4. STUDY POPULATION

4.1 Population (base)

Children aged 0 months to 16 years of age with a primary inguinal hernia will be identified at the outpatient clinic by a (fellow) paediatric surgeon. Parents/caretakers will be asked to participate in this study by the (fellow) surgeon who will also ask informed consent.

4.2 Inclusion criteria

In order to be eligible to participate in this study, a subject must meet all of the following criteria: infants aged 0 months to 16 years of age with a primary unilateral inguinal hernia, undergoing hernia repair.

4.3 Exclusion criteria

A potential subject who meets any of the following criteria will be excluded from participation in this study: Children with 1. incarcerated inguinal hernia, which have to be operated immediately, 2. recurrent hernia 3. ventricular-peritoneal drain, 4. non-descended testis, 5. parents who are not able to understand the nature or consequences of the study.

4.4 Sample size calculation

Reoperation includes both MCIH and recurrence rates. Since recent studies showed that the recurrence rate is similar between the open and laparoscopic group (0.3 vs 0.2% (12)), we used reoperation rates due to MCIH to calculate our sample size. The latest IPEG guidelines states that the MCIH rate after open repair is 6-8%. We expect that the laparoscopic repair reduces the MCIH rate to 1% since the CPPV will be closed during the same procedure. Thus, re-operation based on MCIH is performed in approximately 6% patients in the open group and in approximately 1% in the laparoscopic PIRS group. We consider a reduction of 5% of children requiring reoperation clinically relevant.

A total sample size of 422 patients is needed to detect such a reduction with a power of 0.80 at a two-sided alpha of 0.05 (nQuery Advisor 7.0). Taking into account 10% loss to follow-up we need to include 464 children. Since approximately 800-850 paediatric patients a year undergo inguinal hernia repair in our participating centres, we expect to reach our sample size within one to two years after the start of the trial. After one year of follow-up, we will perform an interim analysis to assess the effect of both treatment strategies.

5. TREATMENT OF SUBJECTS

5.1 Investigational product/treatment

Laparoscopic hernia repair will be performed according the PIRS technique by Patkowski et al (8). This approach will be done under general anaesthesia with muscle relaxation. The patient will be placed in supine position. The operation is as follows: Pneumoperitoneum is established by introducing a 3-5-mm reusable trocar through a sub-umbilical incision. Insufflation pressure is between 8–10 mm Hg. A 30-degree telescope is used and the whole peritoneal cavity is inspected. Any hernia is reduced manually or with the aid of the telescope tip. The location for the needle puncture is assessed by pressing the inguinal ring region from the outside with the tip of a Kocher or any other blunt surgical instrument. Under laparoscopic-guided vision the 16 or 18 gauge injection needle with nonabsorbable thread inside the barrel of the needle is introduced into the body, through the abdominal wall into the abdominal cavity. Needle movement is performed on the outside of the body under laparoscopic vision. With the movements of the tip of the needle the thread passes under the peritoneum, over half of the internal ring including a part of the ligament and adjacent tissue. The thread is pushed through the barrel of the needle into the abdominal cavity and eventually makes a loop. The needle is pulled out, leaving the loop of the thread inside the abdomen. From outside the patient’s body, one of the thread ends is introduced again into the barrel of the needle and the needle passes through the same skin puncture point, to surround the other half of the internal ring with part of the round ligament. To prevent the vas deferens and testicular vessels from injury a small space is left above these structures. The end of the thread goes through the barrel of the needle into the thread loop and the needle is withdrawn. Next, the thread loop is pulled out of the abdomen with the thread end caught by the loop. In this way the thread is placed around the inguinal ring under the peritoneum and both ends exit the skin through the same puncture point. The knot is tied to close the internal ring and is placed under the skin. If a contralateral hernia or CPPV is found, it is closed during the procedure according to the same method as described above, regardless of its diameter. The umbilical wound is closed with absorbable stitches and covered with pressure dressing to prevent hematoma formation. The skin puncture point in the inguinal region is left without any dressing.

5.2 Use of co-intervention (if applicable)

Open hernia correction is as follows: an incision is made in a skin line in the groin. The incision is carried down through the dermis to expose subcutaneous fat and Scarpa fascia. The Scarpa fascia is cut to expose the external oblique muscle. According to the surgeons' preference and/or opinion the external oblique is incised. The hernia sac is searched and if a hernia or PPV is absent, the procedure is finished by closure of Scarpa fascia and skin. If a hernia or PPV is found, the hernia is grasped and cord structures are elevated, creating an opening underneath the sac. An instrument is placed through the opening. Vas deferens and vessels (in boys) are dissected away from the hernia sac or PPV. The sac is then checked for contents. The sac is divided. The proximal sac is then cleaned to the level of the internal inguinal ring, twisted on itself and ligated at the level of the internal ring. The testis (in boys) is returned to the normal scrotal position. Scarpa fascia is closed with a suture. Skin is closed. During the open repair, no contralateral exploration will be performed in the case of a unilateral inguinal hernia.

5.3 Escape medication (if applicable)

Not applicable.

6. INVESTIGATIONAL PRODUCT

Not applicable.

7. NON-INVESTIGATIONAL PRODUCT

Not applicable.

8. METHODS

8.1 Study parameters/endpoints

8.1.1 Main study parameter/endpoint

Number of infants with an ipsilateral recurrent hernia or MCIH after operative repair requiring a reoperation within one year after primary surgery.

8.1.2 Secondary study parameters/endpoints (if applicable)

Operative (i.e. injury of spermatic vessels or spermatic cord, tuba lesions, bleeding and apnea) and postoperative complications (i.e. hematoma/scrotal edema, hydrocele, wound infection, iatrogenic ascent of the testis, testicular atrophy and ileus) within one year after primary surgery.

<u>Definitions</u>

Testicular atrophy: Complete testicular atrophy: no palpable testicular tissue in scrotum.

Partial testicular atrophy: Testis documented to be smaller at follow-up than at time of inguinal hernia repair.

Wound infection/Surgical site infections: Infection, occurring within 30 days of surgery, involving only the skin and subcutaneous tissue. Purulent discharge or organisms isolated from aseptically obtain wound culture must be present. Also at last one of the following signs of infection must be present: Pain or tenderness, localized swelling and redness or heat.

Apnoea: An unexplained episode of cessation of breathing for 20 seconds or longer, or a shorter respiratory pause associated with bradycardia, cyanosis, pallor, and/or marked hypotonia.

Total duration of surgery (including anaesthesia) related to primary open or laparoscopic inguinal hernia repair.

Postoperative pain measured according to the children and infants post-operative pain score (CHIPPS) and VAS and pain medication requirement related to post-operative pain within 2 weeks after primary operation.

Length of hospital stay related to inguinal hernia within one year after primary operation.

Time to full recovery within one year after primary operation.

CPPV rate during laparoscopic contra-lateral exploration in the PIRS group. When seen, the CPPV will be corrected.

MCIH rate within one year after primary operation.

Cosmetic appearance scored on a 5-Likert scale from 1-5 (1= bad, 2=unsatisfactory, 3=satisfactory, 4=good, 5=excellent) within one year after primary operation. Cosmetic appearance will be scored 4 weeks and 1 year after primary surgery.

Cost-effectiveness will be assessed from a societal and health care perspective and will include cost of surgery, hospitalization, control visits, re-operation, complications and loss of productivity of the parents/caretakers. Most data will be registered in the medical records; specific data entry forms will be used. Data on productivity loss will be collected by online questionnaires before surgery, four weeks after surgery, one year after surgery and in case of a recurrence or MCIH, four weeks after re-operation. Cost of the operation will be measured using a bottom-up micro costing approach. Other costs will be valued using guideline prices.

8.1.3 Other study parameters (if applicable)

Not applicable

8.2 Randomisation, blinding and treatment allocation

When parents/caretakers provide informed consent, the child will be randomly assigned (1:1) to the intervention (laparoscopic repair) or control group (open repair) using a web-based application with a computer-generated list with random block sizes of four or six and rendered by an independent data manager. The block size of the randomisation scheme is unknown to the study personnel. Participants and investigators are aware of allocation; masking is impossible because of the nature of the intervention.

8.3 Study procedures

General anaesthesia will be given to all patients undergoing hernia repair. We expect that duration of hospitalization will be similar in both groups, but this is also part of the research questions. Duration of surgery for the laparoscopic technique is expected to be shorter than the open repair group, this is one of the secondary objectives of this study. Different surgical instruments will used during surgery (i.e. telescope, trocars and 18 gauge needle for the laparoscopic procedure). If a disorder of sexual development is found during surgery, the patient will be referred to a paediatrician for further analysis and/or treatment. The children will not be excluded from the study since a disorder of sexual development does not affect our outcome measures or causes extra hazard for the patient.

<u>Assessment of study parameters/endpoints</u>

The following applies to all infants admitted to the hospital for inguinal hernia repair included in this study.

Preoperative size of both testes, the number of operations per infant, length of hospital stay, total duration of operations (including anaesthesia) and perioperative complications of surgery (iatrogenic injury, apnoea) will be recorded in Case Report Forms by the (fellow) paediatric surgeon. One telephone call is performed by a member of the research team after four weeks in the control and intervention group to obtain information regarding recovery of the patient, wound healing and recurrence. Pre-operative and four weeks and one year post-operative, parents or patients older than 12 years of age (with the aid of their parents) will fill out an online questionnaire regarding health of the subject and cost-effectiveness. Invitations for the questionnaires will be send by Castor EDC, in which the results also will be recorded (13).   Furthermore one and two years after surgery telephone call is conducted to asses post-operative complications such as testicular atrophy and recurrence. Prior to the telephone consult, parents are asked to send a photo to the researcher with the help of a secured online application (SURFfilesender) and a coded number

8.4 Withdrawal of individual subjects

Subjects can leave the study at any time for any reason if they wish to do so without any consequences. The investigator can decide to withdraw a subject from the study for urgent medical reasons.

8.4.1 Specific criteria for withdrawal (if applicable)

Not applicable.

8.5 Replacement of individual subjects after withdrawal

After withdrawal patients will not be replaced.

8.6 Follow-up of subjects withdrawn from treatment

Patients withdrawn from treatment will be asked to give permission for a telephone consult one year after surgery to investigate the endpoints of the study.

8.7 Premature termination of the study

The study will be terminated premature if new evidence has arisen that conclude that the effect of the treatment arm is inferior and it is therefore not ethical to continue treatment of more patients with this inferior arm.

In case the study is ended prematurely, the sponsor will notify the accredited METC within 15 days, including the reasons for the premature termination.

9. SAFETY REPORTING

9.1 Temporary halt for reasons of subject safety

In accordance to section 10, subsection 4, of the WMO, the sponsor will suspend the study if there is sufficient ground that continuation of the study will jeopardise subject health or safety. The sponsor will notify the accredited METC without undue delay of a temporary halt including the reason for such an action. The study will be suspended pending a further positive decision by the accredited METC. The investigator will take care that all subjects are kept informed.

9.2 AEs, SAEs and SUSARs

9.2.1 Adverse events (AEs)

Adverse events are defined as any undesirable experience occurring to a subject during the study whether or not considered related to the experimental intervention. Adverse events will be recorded from the start of the study until the moment of first follow-up (at 4 weeks), since adverse events do not occur after a maximum of four weeks after the intervention. All adverse events, within four weeks post-operative, reported spontaneously by the subject or observed by the investigator or his staff will be recorded.

9.2.2 Serious adverse events (SAEs)

A serious adverse event is any untoward medical occurrence or effect that

- results in death;
- is life threatening (at the time of the event);
- requires hospitalisation or prolongation of existing inpatients' hospitalisation;
- results in persistent or significant disability or incapacity;
- is a congenital anomaly or birth defect; or
- any other important medical event that did not result in any of the outcomes listed above due to medical or surgical intervention but could have been based upon appropriate judgement by the investigator.

An elective hospital admission will not be considered as a serious adverse event. Serious adverse events will be recorded and reported throughout the first four post-operative weeks of the study, since adverse advents do not occur after a maximum of four weeks after the intervention.

If a life threatening SAE is encountered, this study will be delayed immediately, since inguinal hernia repair is surgery accompanied by minimal complications. The safety committee is then asked to perform and report an analysis of this SAE before the study will continue

The investigator will report all SAEs to the sponsor without undue delay after obtaining knowledge of the events.

The sponsor will report the SAEs through the web portal *ToetsingOnline* to the accredited METC that approved the protocol, within 7 days of first knowledge for SAEs that result in death or are life threatening followed by a period of maximum of 8 days to complete the initial preliminary report. All other SAEs will be reported within a period of maximum 15 days after the sponsor has first knowledge of the serious adverse events.

9.2.3 Suspected unexpected serious adverse reactions (SUSARs)

Not applicable

9.3 Annual safety report

Not applicable

9.4 Follow-up of adverse events

All AEs that have occurred within the first four post-operative weeks will be followed until they have abated, or until a stable situation has been reached. Depending on the event, follow up may require additional tests or medical procedures as indicated, and/or referral to the general physician or a medical specialist.

SAEs need to be reported till end of study within the Netherlands, as defined in the protocol

9.5 Safety Committee

Both treatment strategies (laparoscopic (intervention) and open (control) hernia repair) are currently performed in children with inguinal hernia who need to undergo repair. Consequently, there are no additional risks for subjects of this study and it is therefore not necessary to install a DSMB. Because this study is undertaken in children, the investigators do want to install a safety committee, which can be asked for help if a SAE is encountered. The committee will be formed by a paediatric surgeon ([redacted]), [redacted]), a surgeon with expertise in hernia repair ([redacted]), and a paediatrician ([redacted]).

10. STATISTICAL ANALYSIS

10.1 Primary study parameter(s)

We will create a 2x2 contingency table with the children that underwent open or laparoscopic repair or not versus the children with and without a ipsilateral recurrent or MCIH hernia in the year after surgery. A chi-square test will be performed and the relative risk, absolute risk difference and odds ratio with their 95% confidence intervals will be calculated. In addition, effect modification will be assessed for gestational age, age at the time of surgery, sex and initial hernia side to identify whether the effect of a treatment is different in groups of patients with different characteristics. Mean differences and corresponding 95% confidence intervals will be calculated for duration of operations (including anaesthesia). Risk differences and 95% confidence intervals will be calculated for complications. Logistic regression analysis will be performed to adjust for possible confounders (i.e. prematurity, side of inguinal hernia), if necessary. All analyses will be performed according to the intention-to-treat principle. Missing values will be analysed using multiple imputation.

10.2 Secondary study parameter(s)

Mean differences and corresponding 95% confidence intervals will be calculated for postoperative pain, duration of hospital admission, time to full recovery and cosmetic appearance. Logistic regression analysis will be performed to adjust for possible confounders (i.e. prematurity, side of inguinal hernia), if necessary. Peri- and post-operative complications will be analysed in a 2x2 contingency table and the data will be assessed by a Chi-Square test. The CPPV rate will only be analysed for the laparoscopic group, since the open repair does not allow for contra-lateral exploration.

10.3 Other study parameters

Cost-effectiveness analyses are performed according to the intention-to-treat principle. For the economic evaluation, missing data are imputed using multiple imputation by chained equations. To deal with the highly skewed nature of cost data, 95%CIs around the differences in costs are estimated using the Bias Corrected and Accelerated Bootstrap method, with 5000 replications. Incremental Cost-Effectiveness Ratios (ICERs) are calculated by dividing the difference in costs by that in QALYs (cost-utility) or by that in ‘disease-specific’ physical functioning (cost-effectiveness). This will be measured by the EQ5D5L questionnaire and an economic costs questionnaire which quantify absenteeism from paid work as well as productivity losses related to unpaid labor. To graphically illustrate the uncertainty surrounding the ICERs, bootstrapped incremental cost-effect

pairs are plotted on cost-effectiveness planes. A summary measure of the joint uncertainty of costs and effects is presented using Cost-Effectiveness Acceptability Curves (CEACs), which indicate the probability of an intervention being cost-effective in comparison with the control condition for a range of willingness-to-pay values (i.e. the maximum amount of money decision-makers are willing to pay to gain one extra unit of effect).

10.4 Interim analysis (if applicable)

An interim analysis will be performed after one year of follow-up to assess the effectivity of the laparoscopic versus open inguinal hernia repair. In case of a significant difference between the open or laparoscopic group, an analysis will be performed to see in the study can continue with a subgroup. If the study can continue with a subgroup, a new sample size calculation will be performed based on the characteristics this subgroup. If no significant difference is found during the interim analysis, the study will continue as stated in this protocol.

11. ETHICAL CONSIDERATIONS

11.1 Regulation statement

The study will be conducted according to the principles of the Declaration of Helsinki (2013) and in accordance with the Medical Research Involving Human Subjects Act (WMO) and Good Clinical Practice.

11.2 Recruitment and consent

All children with an unilateral inguinal hernia visit the outpatient clinic of the (resident/fellow) paediatric surgeon with parents/caretakers. The surgeon explains the study, hands over the information letter and informed consent form to the parents/caretakers, and asks permission to hand over their contact details to the research team. All (fellow) surgeons that will be involved in the conduct of this clinical trial will receive training in order to provide information to the parents/caretakers, according to the SOP and GCP guidelines. Most children are operated within 3-4 weeks after the outpatient clinic visit. After a minimum of seven days, a medical researcher will call the parents/caretakers to ask if they will participate in this study and ask them to sign the two informed consent letters. If, however, the surgery is planned within a week after the outpatient clinic visit, the researcher will call the parents sooner than 7 days, after a minimum of 24 hours, so the parents have enough time to ask questions and think about possible participation. The parents are asked to send the consent letters by post. After receiving the signed informed consent letters, a member of the research team will sign the informed consent letter and randomize the patient using a web-based application. At the day of the surgery, a research team member will collect

the informed consent forms and also signs both forms. One informed consent form will be distributed to the parents/caretakers.

11.3 Objection by minors or incapacitated subjects (if applicable)

This is a therapeutic, intervention study in minors, for which the code of conduct relating to expressions of objection by minors participating in medical research (Netherlands Association for Paediatric Medicine, 2001) is applicable. When a minor resists against the study (which will be difficult to objectivise in these minors), we will not perform the study.

11.4 Benefits and risks assessment, group relatedness

The children in the control group will not benefit directly from this study, except when the results of this study show that open repair is better than the laparoscopic PIRS technique. The risk for these children is that they will develop a contralateral inguinal hernia, which might incarcerate and for which they have to be operated and thus undergo a second operation and anaesthesia.

The patients in the intervention group will undergo laparoscopic repair of the inguinal hernia. There is a possible benefit for these children when a CPPV is present and is repaired during the same procedure.

PIRS repair is only useful in children and not in adults, since paediatric and adult inguinal hernias have a different aetiology. First, in adults an inguinal hernia is a different disorder. In children it is a patent processus vaginalis with normal muscles; in adults tissue degeneration or muscle weakness plays an important role. Therefore mesh reinforcement is needed in adult hernia repair and this is not performed in children. Second, adults are only operated on an inguinal hernia when they experience pain from it (are symptomatic); children are always operated, because of the high risk of incarceration. Third, the risk of developing a contralateral hernia is very low in adults and high (up to 15%) in children. The PIRS technique offers the opportunity to repair a CPPV during the same procedure and thus prevent a MCIH. Fourth, anaesthesia is riskier in children compared to adults. The PIRS procedure allows for contralateral exploration which gives the opportunity to close a CPPV, and thus prevents a second operation and narcosis. Fifth, impact of surgery is larger on social environment of children than in adults. Therefore, this study can only be performed in this group of minors.

11.5 Compensation for injury

The sponsor/investigator has a liability insurance which is in accordance with article 7 of the WMO.

The sponsor (also) has an insurance which is in accordance with the legal requirements in the Netherlands (Article 7 WMO). This insurance (Onderlinge Waarborgmaatschappij Centramed B.A. PO Box 7374, 2701 AJ Zoetermeer) provides cover for damage to research subjects through injury or death caused by the study.
The insurance applies to the damage that becomes apparent during the study or within 4 years after the end of the study. The sponsor (also) has an insurance, which is in accordance with the legal requirements in the Netherlands (Article 7 WMO). This insurance provides cover for damage to research subjects through injury or death caused by the study.

11.6 Incentives (if applicable)

Not applicable.

12. ADMINISTRATIVE ASPECTS, MONITORING AND PUBLICATION

12.1 Handling and storage of data and documents

To ensure their privacy, all the participants' data will be encoded using a five-digit number that is only used for this study (XX-XXX). This encoding will be as follows: the first two digits are for the participating centre; the second three digits are for the consecutive patient number in that centre. Only the principal investigator, the coordinating investigator and the project leader will have access to the encoding key which links the code to the participants' personal data. After termination of the study, participants will have full access to their results and data will be explained to them when requested from the researcher at their site. This researcher can obtain the results via the coordinating investigator or the project leader. In case this study (by coincidence) reveals any findings important to participants, the principal investigator makes sure the attending doctor will be informed.

The start date of this study will be reported to the METc. All collected data will be stored at Amsterdam University Medical Center, location AMC, for a maximum of 15 years.

We will collect following baseline characteristics and data from the participants: inguinal hernia in first degree siblings, gestational age, birth weight, age at day of surgery, weight at day of surgery, duration of surgery, duration of hospital stay, sex, side of the hernia, comorbidity, CPPV rate, complications (wound infection, testicular atrophy, apnoea, recurrence), development of contralateral hernia.

12.2 Monitoring and Quality Assurance

VU Medical Centre provides an independent monitor who will perform interim monitoring. The monitor will verify that the rights and well-being of patients are protected, the reported trial data are accurate, complete, and verifiable from source documents and the conduct of the trial is in compliance with the currently approved protocol/amendment(s), with Good Clinical Practice (GCP) and with the applicable regulatory requirement(s). Direct access to source documentation (medical records) must be allowed for the purpose of verifying that the data recorded in the Case Report Forms is consistent with the original source data. Intensity of this verification will be assessed by risk classification of this study. In any case the monitor will review the in-and exclusion criteria, primary endpoints and correct reporting of (S)AEs and SUSARs according to applicable laws and regulations. The sponsor expects that during monitoring visits the relevant investigational staff will be available, the source documentation (including informed consents) will be available and a suitable environment will be provided for review of study-related documents.

12.3 Amendments

Amendments are changes made to the research after a favourable opinion by the accredited METC has been given. All amendments will be notified to the METC that gave a favourable opinion.

12.4 Annual progress report

The sponsor/investigator will submit a summary of the progress of the trial to the accredited METC once a year. Information will be provided on the date of inclusion of the first subject, numbers of subjects included and numbers of subjects that have completed the trial, serious adverse events/ serious adverse reactions, other problems, and amendments.

12.5 Temporary halt and (prematurely) end of study report

The investigator/sponsor will notify the accredited METC of the end of the study within a period of 8 weeks. The end of the study is defined as the last patient's last visit.

The sponsor will notify the METC immediately of a temporary halt of the study, including the reason of such an action.

In case the study is ended prematurely, the sponsor will notify the accredited METC within 15 days, including the reasons for the premature termination.

Within one year after the end of the study, the investigator/sponsor will submit a final study report with the results of the study, including any publications/abstracts of the study, to the accredited METC.

12.6 Public disclosure and publication policy

All results, positively or negatively, will be presented at (inter)national conferences and published in peer reviewed (inter)national journals. In case of rejection of results by peer reviewed journals, the results will be made public in a trial registry or a database. A clinical trial agreement will be signed by involved parties. The study will be registered as a clinical trial in a public trial registry before the first patient is recruited.

13. STRUCTURED RISK ANALYSIS

Not applicable

14. Appendix 1

[redacted]

[redacted]

[redacted]

[redacted]

[redacted]

[redacted]

[redacted]

[redacted]

[redacted]

[redacted]

[redacted]

[redacted]

REFERENCES

1. Burgmeier C, Dreyhaupt J, Schier F. Comparison of inguinal hernia and asymptomatic patent processus vaginalis in term and preterm infants. J Pediatr Surg. 2014;49(9):1416-8.
2. Chang SJ, Chen JY, Hsu CK, Chuang FC, Yang SS. The incidence of inguinal hernia and associated risk factors of incarceration in pediatric inguinal hernia: a nation-wide longitudinal population-based study. Hernia. 2016;20(4):559-63.
3. Abdulhai SA, Glenn IC, Ponsky TA. Incarcerated Pediatric Hernias. The Surgical clinics of North America. 2017;97(1):129-45.
4. Esposito C, Giurin I, Alicchio F, Farina A, Iaquinto M, Scermino S, et al. Unilateral inguinal hernia: laparoscopic or inguinal approach. Decision making strategy: a prospective study. Eur J Pediatr. 2012;171(6):989-91.
5. Walther-Larsen S, Rasmussen LS. The former preterm infant and risk of post-operative apnoea: recommendations for management. Acta anaesthesiologica Scandinavica. 2006;50(7):888-93.
6. Andropoulos DB, Greene MF. Anesthesia and Developing Brains - Implications of the FDA Warning. The New England journal of medicine. 2017;376(10):905-7.
7. Dreuning K, Maat S, Twisk J, van Heurn E, Derikx J. Laparoscopic versus open pediatric inguinal hernia repair: state-of-the-art comparison and future perspectives from a meta-analysis. Surg Endosc. 2019.
8. Patkowski D, Czernik J, Chrzan R, Jaworski W, Apoznanski W. Percutaneous internal ring suturing: a simple minimally invasive technique for inguinal hernia repair in children. J Laparoendosc Adv Surg Tech A. 2006;16(5):513-7.
9. Shibuya S, Fujiwara N, Ochi T, Wada M, Takahashi T, Lee KD, et al. The learning curve of laparoscopic percutaneous extraperitoneal closure (LPEC) for inguinal hernia: protocoled training in a single center for six pediatric surgical trainees. BMC Surg. 2019;19(1):6.
10. Barroso C, Etlinger P, Alves AL, Osorio A, Carvalho JL, Lamas-Pinheiro R, et al. Learning Curves for Laparoscopic Repair of Inguinal Hernia and Communicating Hydrocele in Children. Front Pediatr. 2017;5:207.
11. Feng S, Zhao L, Liao Z, Chen X. Open Versus Laparoscopic Inguinal Herniotomy in Children: A Systematic Review and Meta-Analysis Focusing on Postoperative Complications. Surg Laparosc Endosc Percutan Tech. 2015;25(4):275-80.
12. Nakashima M, Ide K, Kawakami K. Laparoscopic versus open repair for inguinal hernia in children: a retrospective cohort study. Surgery today. 2019.
13. Castor EDC. Castor Electronic Data Capture 2019 [27 Aug. 2019]. Available from: https://castoredc.com.
