# Supplementary material for: HERNIIA-II trial (Hernia Endoscopic oR opeN repair In chIldren Analysis): a protocol of a multicentre randomised controlled trial to study the (cost-)effectiveness of laparoscopic hernia repair compared to open hernia repair in children 0–16 years
Source: BMJ Open. 2025 Dec 4;15(12):e110662. doi: 10.1136/bmjopen-2025-110662 (PMC12684125; doi:10.1136/bmjopen-2025-110662)
Supplement: Supplementary file 4 [file bmjopen-2025-110662.reviewer_comments.pdf]

PEER REVIEW HISTORY

BMJ Open publishes all reviews undertaken for accepted manuscripts. Reviewers are asked to complete a checklist review form and are provided with free text boxes to elaborate on their assessment. These free text comments are reproduced below.

ARTICLE DETAILS

Title (Provisional)

The HERNIIA-II trial ('Hernia Endoscopic oR opeN repair In chIldren Analysis"): A protocol of a randomized controlled trial to study the (cost-) effectiveness of laparoscopic hernia repair compared to open hernia repair

Authors

Maat, Sanne C.; de Vreeze, Lore Elise; Eurlings, Roxanne; Anema, Johannes; Van Baren, Robertine; Been, Jasper V.; van den Broek, Frank; Cakir, Hamit; van Dongen, Johanna M.; Ferenschild, Floris; de Graaf, Jurgen; Nijveldt, Robert; Ottenhof, Anne; Ploeg, Arianne J; Rippen, Hester; Ruiterkamp, Jetske; Twisk, Jos W.R.; Vermeulen, Erik; De Wit, Ralph; Zijp, Gerda; van Heurn, Ernest L.W.; Derikx, Joep P.M.

This article was not externally reviewed at BMJ Open. Protocols that have been independently assessed prior to submission to BMJ Open are usually fast-tracked to publication on the grounds that further substantial changes will not be possible. This independent assessment will usually be external, independent review for both a substantial grant award from a non-commercial or government funder and ethics approval.
